# Discovery of Novel Insulin Sensitizers: Promising Approaches and Targets

**DOI:** 10.1155/2017/8360919

**Published:** 2017-06-04

**Authors:** Yadan Chen, Haiming Ma, Dasheng Zhu, Guowei Zhao, Lili Wang, Xiujuan Fu, Wei Chen

**Affiliations:** ^1^Department of Pharmacy, The Second Hospital of Jilin University, Changchun 130041, China; ^2^Department of Pharmacy, China-Japan Union Hospital of Jilin University, Changchun 130041, China; ^3^Department of Pharmacy, Beijing Boai Hospital, China Rehabilitation Research Center, Beijing 100068, China; ^4^Beijing Institute of Pharmacology and Toxicology, Beijing 100850, China

## Abstract

Insulin resistance is the undisputed root cause of type 2 diabetes mellitus (T2DM). There is currently an unmet demand for safe and effective insulin sensitizers, owing to the restricted prescription or removal from market of certain approved insulin sensitizers, such as thiazolidinediones (TZDs), because of safety concerns. Effective insulin sensitizers without TZD-like side effects will therefore be invaluable to diabetic patients. The specific focus on peroxisome proliferator-activated receptor *γ*- (PPAR*γ*-) based agents in the past decades may have impeded the search for novel and safer insulin sensitizers. This review discusses possible directions and promising strategies for future research and development of novel insulin sensitizers and describes the potential targets of these agents. Direct PPAR*γ* agonists, selective PPAR*γ* modulators (sPPAR*γ*Ms), PPAR*γ*-sparing compounds (including ligands of the mitochondrial target of TZDs), agents that target the downstream effectors of PPAR*γ*, along with agents, such as heat shock protein (HSP) inducers, 5′-adenosine monophosphate-activated protein kinase (AMPK) activators, 11*β*-hydroxysteroid dehydrogenase type 1 (11*β*-HSD1) selective inhibitors, biguanides, and chloroquines, which may be safer than traditional TZDs, have been described. This minireview thus aims to provide fresh perspectives for the development of a new generation of safe insulin sensitizers.

## 1. Introduction

According to the World Health Organization, the morbidity of diabetes mellitus (DM), which is a global noninfectious epidemic disease, is expected to continue to rise in the coming decades [1]. The surge in type 2 DM (T2DM) that affects more than 90% of diabetic patients can be attributed to the increasing prevalence of insulin resistance (IR) in the general population, which is partially triggered by the widespread occurrence of obesity and metabolic syndrome [2]. IR, which has been considered a root cause of T2DM, is a pathological state in which the target cells in liver, skeletal muscle, and adipose tissue fail to respond properly to insulin, resulting in their inability to efficiently uptake and metabolize glucose [3–5]. A comprehensive review of insulin receptor signaling has been presented previously [4]. IR leads to a loss of response from the peripheral insulin target tissues (mainly the liver, adipose, and muscle tissues) to insulin. Additionally, IR may impair the ability of the pancreatic islets to synthesize and secrete sufficient insulin to address the metabolic needs of the body. The clinical manifestations of IR include hyperinsulinemia, hyperglycemia, hyperlipidemia, increased circulating inflammatory marker levels, and diminished plasma adiponectin levels. Several factors, including defective insulin signal transduction, impaired effectors within insulin-dependent pathways, and enhanced insulin-antagonizing pathways, contribute to the etiology of IR at the cellular level [4]. Thus, it is apparent that T2DM patients urgently require drugs that target the etiology of the disease rather than those that merely ameliorate the symptoms.

Although several mechanism-based drugs, such as dipeptidyl peptidase 4 (DPP-4) inhibitors, and sodium/glucose cotransporter 2 (SGLT2) inhibitors, have been introduced in the market during the past decade for the treatment of T2DM and/or DM-related diseases, none of them specifically target IR except for insulin sensitizers such as thiazolidinediones (TZDs), which are peroxisome proliferator-activated receptor *γ* (PPAR*γ*) agonists. Insulin sensitizers, which exert positive effects on both insulin target tissues and the pancreas, can potentially reverse the course of the disease and prevent the progression to diabetic complications. Hence, insulin sensitizers would undoubtedly be preferable for T2DM patients, if their side effects, such as fluid retention, weight gain, hemodilution, edema, and congestive heart failure, could be minimized. However, a safe and effective insulin sensitizer remains elusive, as the specific focus on PPAR*γ*-based agents in the past may have partially hampered our efforts towards finding such novel and safer insulin sensitizers. Therefore, this review focuses on recent advances in understanding the pathophysiological mechanisms of IR and describes the PPAR*γ* targets of the classical insulin sensitizers and beyond PPAR*γ*, some newly discovered targets.

## 2. Classification of New-Generation Insulin Sensitizers

**Figure 1:**
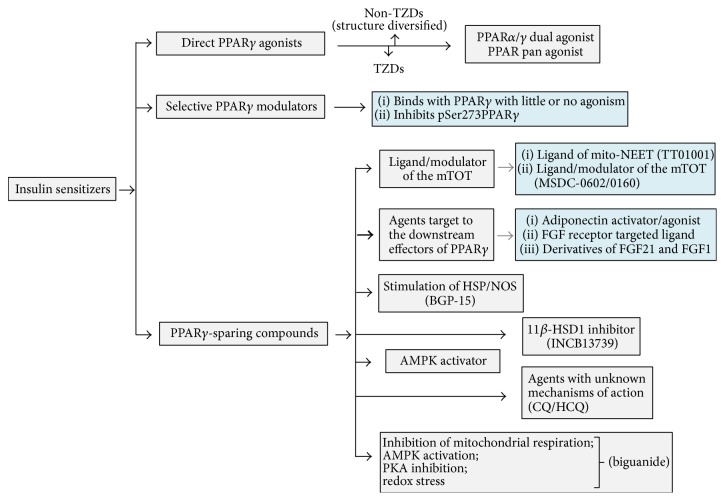
*Classification of new-generation insulin sensitizers based on their mechanisms or targets*. AMPK, adenosine monophosphate-activated protein kinase; CQ, chloroquine; FGF, fibroblast growth factor; HCQ, hydroxychloroquine; 11*β*-HSD1, 11*β*-hydroxysteroid dehydrogenase type 1; HSP, heat shock protein; mTOT, mitochondrial target of TZDs; mitoNEET, mitochondrial Asn-Glu-Glu-Thr; NOS, nitric oxide synthase; PKA, protein kinase A; PPAR*γ*, peroxisome proliferator-activated receptor *γ*; TZDs, thiazolidinediones.

The new-generation insulin sensitizers may be broadly classified as direct PPAR*γ* agonists, selective PPAR*γ* modulators, and PPAR*γ*-sparing compounds (Figure 1). Direct PPAR*γ* agonists include the pure PPAR*γ* agonists and PPAR*α*/*γ* dual or PPAR pan agonists. Selective PPAR*γ* modulators are mainly compounds that bind with PPAR*γ* but exhibit little or no agonism and instead inhibit PPAR*γ* phosphorylation at serine 273 in a tissue-selective manner [6]; PPAR*γ*-sparing compounds include those that do not bind with PPAR*γ* but bind with the newly identified mitochondrial targets of TZDs, that is, the mitochondrial outer or inner membrane proteins [7, 8], compounds that target the downstream effectors of PPAR*γ*, such as the adiponectin and fibroblast growth factor 21 (FGF21) signaling pathways, heat shock protein (HSP) inducers, 5′ adenosine monophosphate-activated protein kinase (AMPK) activators, 11*β*-hydroxysteroid dehydrogenase type 1 (11*β*-HSD1) inhibitors, and molecules such as biguanides and chloroquines (CQs), whose molecular targets or mechanisms are still not completely understood.

## 3. Direct PPAR*γ* Agonists

### 3.1. Pure/Selective PPAR*γ* Agonists

**Figure 2:**
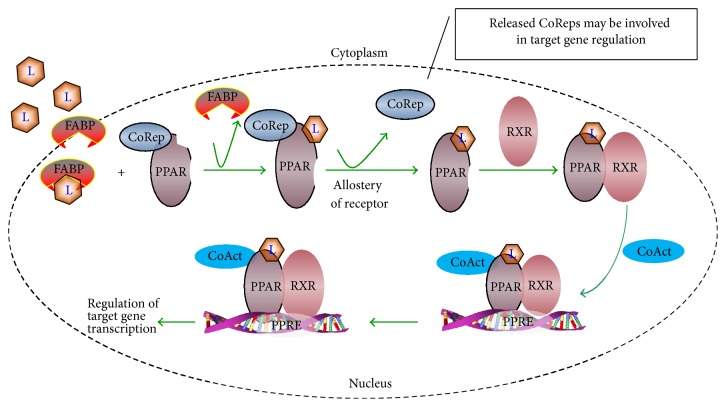
*Schematic representation of the mechanism of PPARγ agonist signaling*. L, ligand (including natural ligand and synthetic PPAR*γ* agonist); CoRep, corepressor; CoAct, coactivator; FABP, fatty acid-binding protein; PPAR, peroxisome proliferator-activated receptor; RXR, retinoid X receptor; PPRE, PPAR*γ* response elements.

PPAR*γ* is a member of the nuclear hormone receptor (NR) superfamily and belongs to the NR1C subgroup [9]. It is predominantly expressed in the adipose tissue and in low levels in the liver, muscles, and other tissues [6]. TZDs, which are pure PPAR*γ* full agonists, have been widely used to treat T2DM for nearly 20 years and are referred to as the classical “insulin sensitizers,” as they act to restore blood glucose to normal levels by increasing the insulin sensitivity of target tissues without the risk of causing hypoglycemia, unlike agents such as secretagogues. The mechanism of action of TZDs involves regulating the expression of a panel of PPAR*γ* downstream target genes associated with glucose and lipid metabolism, adipokines secretion, and inflammatory reactions in target tissues [9, 10]. PPAR*γ* signaling is initiated by the formation of a heterodimer between PPAR*γ* and retinoid X receptor *α* (RXR*α*) after the binding of the ligand or agonist to the ligand-binding domain (LBD) of PPAR*γ* and the subsequent dissociation of its corepressors [9, 11]. The PPAR*γ*-RXR*α* complex then recruits specific coactivators depending on the tissue and cellular environment [6] and regulates gene transcription by binding to specific PPAR*γ* response elements (PPREs) within the promoter region of the target genes (Figure 2). The roles of PPAR*γ* in human and mouse physiology have been reviewed in depth previously [11].

Troglitazone (Rezulin®), the first oral TZD approved in 1997, was discovered before PPAR*γ* was identified as a target of insulin sensitizers. The confirmation of PPAR*γ* as the primary molecular target of TZDs in the mid-1990s spurred the search for novel antidiabetic drugs with stronger PPAR*γ* agonism, although troglitazone was removed from the market in 2001, owing to severe hepatotoxicity [12]. Subsequently, rosiglitazone and pioglitazone, two other pure PPAR*γ* full agonists, were successfully introduced into the market in 1999 for the treatment of T2DM. There was no evidence of hepatotoxicity in clinical trials for either rosiglitazone or pioglitazone [13]. The treatment of T2DM with these drugs was a step toward targeting IR, which is the etiology of the disease, rather than merely promoting insulin release from islet. Therefore, TZDs could potentially rescue the pancreatic islets from the burden of synthesizing and secreting more insulin and consequent functional exhaustion. Although these advantages had resulted in TZDs becoming one of the best-selling drugs in the world for over 10 years, the successive disclosure of adverse events, such as increased cardiovascular risk, fluid retention, bone fractures, hepatotoxicity, and body weight gain, from 2005 onwards posed a severe threat to the clinical prescription of the classical insulin sensitizers [14–16]. Rosiglitazone has been withdrawn by the European Medicines Agency, and its prescription has been restricted by the United States Food and Drug Administration (FDA) [16]. Further, the only available TZD, pioglitazone, carries a black-box warning on the label for potential cardiovascular risks and increased risk of bladder cancer [17]. Thus, the clinical use of first-generation pure PPAR*γ* full agonists has been greatly restricted.

### 3.2. From Pure PPAR*γ* Agonists to PPAR*α*/*γ* Dual or PPAR Pan Agonists

In addition to PPAR*γ*, the NR1C subgroup includes two other members, namely, PPAR*α* and PPAR*β*/*δ*, which also modulate the expression of a series of target genes that play pleiotropic roles in regulating glucose, lipid, and cholesterol metabolism. These characteristics have made the three PPAR subtypes attractive therapeutic targets for developing novel drugs against T2DM and other metabolic diseases. PPAR*α* agonists (fibrates), which are currently being marketed as effective hypolipidemic agents that decrease the progression of atherosclerosis, have also been found to improve glucose intolerance in T2DM animals and patients [18, 19]. These findings suggest that the simultaneous activation of both PPAR*α* and PPAR*γ* using a single molecule may combine the advantages of PPAR*α* and PPAR*γ* agonism and avoid some of the disadvantages of pure PPAR*γ* agonists [20]. The development of such potent PPAR*α*/*γ* dual agonists or PPAR pan agonists as insulin sensitizer candidates was hotly pursued by global pharmaceutical companies from 1998 to 2006, with the expectation of providing a broad spectrum of beneficial metabolic effects [21, 22]. However, the unpredictable side effects, such as carcinogenesis and cardiovascular adverse events, of these newly reported PPAR*α*/*γ* dual agonists in clinical trials discouraged researchers and almost led to the termination of basic and clinical research on these drugs. Nevertheless, the potential to develop multitargeted PPAR*α*/*γ*/*δ* pan agonists as antidiabetic or hypolipidemic drugs is still being actively investigated in some locations [23–26].

## 4. Selective PPAR*γ* Modulators (sPPAR*γ*Ms)

As genetic and epidemic studies indicated that the side effects of TZDs were associated with the overactivation of PPAR*γ*, and physiologically appropriate PPAR*γ* activity was beneficial for reducing IR and other T2DM-related risks, partial PPAR*γ* agonists were extensively researched for a short period. Choi et al. reported in 2010 that cyclin-dependent kinase 5- (CDK5-) stimulated phosphorylation at serine 273 of PPAR*γ* (pSer273PPAR*γ*), which led to the dysregulated expression of a set of genes in adipose tissue, especially the epididymal white fat tissue (eWAT), was the critical link between obesity and IR, and pSer273PPAR*γ* inhibition rather than PPAR*γ* agonism was responsible for the insulin-sensitizing and antidiabetic effects of PPAR*γ* agonists [27, 28]. Thus, these distinctive properties of PPAR*γ* suggested the possibility of selectively modulating it such that beneficial therapeutic effects could be attained without the unwanted effects of traditional PPAR*γ* full agonists.

The compounds discovered based on this concept have been variously termed selective PPAR*γ* modulators (sPPAR*γ*Ms), partial PPAR*γ* agonists, or nonagonist PPAR*γ* ligands in the literature. In this review, we uniformly refer to them as sPPAR*γ*Ms. Unlike classical PPAR*γ* full agonists, sPPAR*γ*Ms bind to the LBD of PPAR*γ* in various ways via an activation function 2 motif (AF2) that displays great flexibility in response to diverse ligands, resulting in different receptor conformations and coactivator and/or corepressor recruitment in different tissues [6]. Thus, sPPAR*γ*Ms selectively regulate the expression of genes that are responsible for insulin sensitization, such as the adiponectin gene, without affecting the transcription of genes related to weight gain, adipogenesis, and fluid retention. Several sPPAR*γ*Ms, such as UHC1 (modified from SR1664), CMHX008, INT131, balaglitazone, and L312 have been successfully developed by various institutions [29–33] and demonstrated to be potent insulin sensitizers with similar antidiabetic effects as TZDs and better safety profiles, with decreased incidence of TZD-like adverse effects such as heart failure, peripheral edema, and myocardial infarction. These studies suggest that focusing on sPPAR*γ*Ms presents a great opportunity for developing antidiabetic drugs that offer the desired efficacy of PPAR*γ* agonists without some of their potential adverse effects, although it remains a challenging endeavor. Additionally, it is critical to investigate the different conformations of PPAR*γ* in the presence of distinct ligands, as the ligands interact in different ways with the receptor, recruit different coactivators/corepressors, and exhibit different interactions with the response element, thus triggering the transcription of diverse genes. This may even make it possible to accurately predict the effects of a particular agent.

## 5. PPAR*γ*-Sparing Compounds

### 5.1. Ligands/Modulators of the Mitochondrial Targets of TZDs

Several recent studies have proposed that PPAR*γ* could not be completely responsible for the insulin-sensitizing efficacy of the classical TZDs [7, 34, 35]. The identification of a novel binding site for TZDs in the mitochondrial membrane has instead suggested the presence of an alternative, PPAR*γ*-independent mode of action for this class of TZD drugs, which in turn signals the possibility of rationally designing insulin sensitizers that are distinct from PPAR-activating compounds.

#### 5.1.1. MitoNEET (or CDGSH1) Ligands

MitoNEET (mitochondrial Asn-Glu-Glu-Thr), an iron-sulfur- (Fe-S-) containing mitochondrial outer membrane protein that is involved in transferring the Fe-S clusters to the cytosolic aconitase [36], had been identified by several research groups as a binding target of the insulin sensitizer pioglitazone [37–39]. A potential ligand-binding site, which was identified on the surface of both protomers of the mitoNEET homodimer via blind docking using AutoDock Vina and in vitro fluorescence binding assays, was used as the basis for generating a number of structurally diverse, novel mitoNEET ligands that were structurally distinct from TZDs [39]. Similarly, a mitoNEET ligand, TT01001, which was designed based on the structure of pioglitazone, was reported to show the same potency as pioglitazone in improving hyperglycemia, hyperlipidemia, and glucose intolerance in diabetic (db/db) mice by significantly suppressing the elevated mitochondrial complex activity of skeletal muscles, without activating PPAR*γ* or causing weight gain [40]. MitoNEET has thus been considered a potential target in the treatment of T2DM. Although further studies are required to thoroughly enumerate the physiological role of mitoNEET in regulating the mitochondrial function and its role in DM, current research indicates that alteration of mitochondrial function via mitoNEET may be a valuable insulin-sensitizing strategy.

#### 5.1.2. Mitochondrial Target of TZDs (mTOT) Protein Complex Modulators

As the cross-linking of mitochondrial membranes by TZD probes was observed to occur even in the complete absence of mitoNEET, a study published in 2013 found that mitoNEET was not the primary mitochondrial TZD target [7]. Further studies using radiolabeled TZD probes identified mitochondrial pyruvate carrier 2 (Mpc2 or BRP44), a different protein similar in size to mitoNEET in the inner mitochondrial membrane, as the true binding partner of TZDs. TZDs can rapidly suppress glucose production in perfused livers or isolated hepatocytes and increase glucose uptake in myocytes (within 90 min of TZD treatment) directly by specifically inhibiting Mpc-mediated pyruvate transport into the mitochondria [35, 41]. Mpc, formed by two paralogous subunits, Mpc1 and Mpc2, serves as the channel to facilitate pyruvate transport across the impermeable mitochondrial membrane [8]. Knockdown of Mpc1 or Mpc2 in flies or preincubation with a specific pyruvate transport inhibitor (UK5099) can block the cross-linking of mitochondrial membranes by TZD probes [7]. Importantly, UK5099 can reproduce the effects of TZDs on glucose uptake and hepatic gluconeogenesis from pyruvate [35, 42]. Moreover, mice lacking hepatic MPC2 display impaired hepatic mitochondrial pyruvate metabolism and gluconeogenesis [43]. These data strongly suggest that mild MPC inhibition can be insulin-sensitizing. The newly discovered mTOT should contain at least Mpc1 and Mpc2 as part of a multisubunit complex, along with other unidentified proteins that may vary depending on the cell type [7, 35]. It has also been suggested that mTOT should be viewed as a sensor that signals the coordination multiple parallel pathways in different cells or tissues to increase insulin response and fatty acid oxidation [7, 44, 45].

Subsequently, two new representative mTOT modulators, MSDC-0602 and MSDC-0160, were developed based on this concept by the Metabolic Solutions Development Company in 2013 [45], and phases IIa and IIb trials were completed [46]. Although the PPAR*γ* binding and activating ability of these modulators were much weaker than those of pioglitazone, they displayed equivalent efficacy in managing hyperglycemia in T2DM patients and caused significantly lower fluid retention. Additionally, MSDC-0160 could correct the dysregulated gene expression observed in flies with high-sucrose diet-induced DM [7]. Further, knockdown of the Mpc1 ortholog in flies, which reduced the expression of both Mpc1 and Mpc2, prevented all responses to MSDC-0160 [45]. These data support the hypothesis that the PPAR*γ* binding and activating ability can be uncoupled from the insulin-sensitizing ability. The mitochondrial protein complex, mTOT, thus provides a new target for rationally designing PPAR*γ*-spared insulin sensitizers. However, until the precise molecular target of TZDs is finally identified, utilizing a network pharmacology approach aimed at understanding the comprehensive mechanisms of action of TZDs in improving IR and managing DM appears more reasonable.

### 5.2. Insulin Sensitizers That Target the Downstream Effectors of PPAR*γ*

#### 5.2.1. Adiponectin Receptor (AdipoR) Activators/Agonists

Adipokines are pleiotropic molecules that play multiple roles in metabolic and inflammatory responses [47]. In recent years, most research efforts have been focused on studying diabetes associated insulin-sensitizing adipokines, such as adiponectin (also known as Acrp30), vaspin visfatin, metrnl (also known as subfatin), and retinol binding protein 4 (RBP4). [47–50]. Their pathophysiological roles are being actively explored. The adipokine itself or its mimics or derivatives may become a therapeutic target for diabetes and underlying disturbances.

Adiponectin, an adipokine secreted by adipocytes, was originally identified in 1995 [51] and exists as low-, medium-, and high-molecular weight (LMW, MMW, and HMW) oligomers. While each isoform of adiponectin has distinct biological functions, the HMW oligomer is the most biologically active form with respect to glucose homeostasis and possesses the most potent insulin-sensitizing activity. Research over the past two decades suggests that adiponectin plays an important role in the development of IR [47, 52]. Circulating levels of adiponectin were notably reduced (hypoadiponectinemia) under conditions of obesity, IR, and T2DM, whereas overexpression or administration of adiponectin improved overall insulin action and reversed hyperglycemia in obese mice, independent of plasma insulin levels [52, 53]. Although adiponectin by itself appears to be an effective insulin enhancer, the short half-life (32 min for trimers and 83 min for HMW and MMW proteins [54]) and other limitations have hindered its use as a therapeutic drug. Adiponectin is also considered to play a role in the functioning of other insulin sensitizers, as its levels are reportedly increased by TZD treatment, and the insulin-sensitizing effect of TZDs tends to be impaired in adiponectin-deficient mice [55]. Increased adiponectin levels also promote the PPAR*γ* pathway and form a positive feedback loop that comprehensively addresses the cause of IR and DM.

Adiponectin predominantly binds to the AdipoR1 and AdipoR2 receptors [56], which are abundantly expressed in the skeletal muscle and liver, respectively [57]. These receptors regulate metabolic gene expression and insulin sensitivity in insulin target tissues and play important roles in the development of IR and DM. The reduced expression of AdipoRs in obesity and DM is correlated with a decrease in the effect of adiponectin [52]. Adiponectin exerts its antidiabetic effects by binding to the two AdipoRs and activating the PPAR*α* and AMPK pathways, with APPL1 (adaptor protein, phosphotyrosine interacting with PH domain and leucine zipper 1) as the key adaptor that directly binds to the intracellular domains of AdipoRs via its C-terminal phosphotyrosine binding (PTB) and coiled-coil (CC) domains. This leads to enhanced fatty acid oxidation and decreased hepatic glucose production, which is independent of insulin levels or glucose disposal rate in peripheral tissues [52, 56, 58]. A more comprehensive review of adiponectin signaling has been presented previously [52, 59]. In contrast, the disruption of both AdipoR subtypes has been found to abolish the effects of adiponectin, resulting in elevated lipid accumulation in insulin target issues (mainly the liver and skeletal muscles) and finally leading to IR [56].

Recently, the first orally active AdipoR activator, AdipoRon, which binds to both AdipoRs and ameliorates IR and glucose intolerance in high-fat diet-induced obese mice in an AdipoR-dependent manner, was discovered [60]. AdipoRon has also been shown to reverse the diabetic condition of genetically diabetic db/db mice and prolong their lifespan [61]. Additionally, it exerted cardioprotective effects against postischemic cardiac injury, a common DM-related cardiovascular complication [62]. However, the low affinity of AdipoR for the currently available small molecule activators is an unresolved challenge. Thus, based on current studies, the AdipoR signaling pathway remains a potential pharmacological target for IR and T2DM, and adiponectin derivatives or orally active small molecules with higher affinity to AdipoR may give rise to novel insulin sensitizers for the treatment of T2DM in the near future.

#### 5.2.2. Fibroblast Growth Factor 21 (FGF21) and Its Derivatives

FGF21, a member of the endocrine FGF subfamily, has been identified as a PPAR*γ* target in the adipose tissue. As a hormone that plays pleiotropic roles in regulating glucose and lipid homeostasis, insulin sensitivity, and cellular oxidative stress, its glucose-lowering effect has been well demonstrated in various diabetic mouse and primate models [63–67]. Recently, FGF1, the prototype of the 22-member FGF family, was found to be transcriptionally regulated by PPAR*γ* in adipose tissue, and the PPRE was identified within the FGF1 gene [65, 68]. FGF1 knockout mice developed severe IR and an aggressive diabetic phenotype when challenged with a high-fat diet [68]. Recombinant FGF1 (rFGF1) administered by injection dose-dependently ameliorated insulin insensitivity and hyperinsulinemia, normalized glucose levels, and increased the hepatic glycogen content in genetically diabetic (ob/ob and db/db) and high-fat diet-induced obese insulin-resistant mice, with no hypoglycemia even at higher doses [69]. Further, there was no resistance to the treatment in ob/ob mice, when the treatment was extended over 1 month. The FGF1-mediated whole-body insulin sensitization was attributed to a simultaneous increase in insulin-dependent glucose uptake in target tissues and suppression of hepatic glucose production.

Studies by Lin et al. [70] revealed that the insulin-sensitizing adipokine, adiponectin, was a downstream effector of FGF21 that coupled the effects of FGF21 in local adipocytes to the liver and skeletal muscles, thus mediating the systemic effects of FGF21 on energy metabolism and insulin sensitivity. Significantly, these benefits were not accompanied by side effects, such as weight gain, fluid retention, and bone loss that are commonly associated with TZDs, the classical insulin sensitizers. Moreover, FGF1 did not stimulate insulin release from the pancreatic islets or possess insulin-mimetic effects but could markedly enhance the glucose-lowering effects of exogenously supplied insulin in streptozotocin-induced type 1 DM (T1DM) mouse models [69], without affecting the blood glucose and insulin levels in chow-fed normoglycemic mice [68]. Similarly, FGF21 increased insulin content and secretion in diabetic islets and protected the pancreatic *β* cells from apoptosis by activating the extracellular signal-regulated kinase 1/2 (ERK1/2) and Akt signaling pathways [71]. These recent findings collectively indicate that FGF1 and FGF21 possess all the characteristics of a good insulin sensitizer without TZD-like adverse effects.

Other studies have found that FGF1 may be exerting its insulin-sensitizing activity through the FGF1 receptor (FGFR1) in adipose tissue, although it can bind and activate all alternatively spliced forms of the four tyrosine kinase FGF receptors (FGFR1–FGFR4). rFGF1 and rFGF^ΔDNT^, which are FGF1 ligands generated by removing the first 24 residues from the amino terminus of FGF1, lost their glucose-lowering effect on adipose tissue, specifically in FGFR1 knockout mice [72]. In addition, LY2405319, an engineered FGF21 variant, has recently been found to be effective in alleviating IR in diabetic mice and monkeys [73, 74]. Thus, we can tentatively conclude that FGF receptor-targeted ligands or derivatives of FGF21 and FGF1 are promising insulin sensitizer candidates for fighting T2DM. However, as with all protein-based therapeutics, some major hurdles, such as a short half-life, enzymatic degradation, and low bioavailability, still need to be overcome, and further clinical studies are required to verify whether these candidates can act as effective antidiabetic agents in humans.

### 5.3. HSP/Nitric Oxide Synthase (NOS) Inducers

BGP-15 [O-(3-piperidino-2-hydroxy-1-propyl)-nicotinic acid], an HSP/chaperone inducer that was originally developed as a chemoprotectant, has been identified as a new type of insulin sensitizer with a novel pharmacological mechanism [75, 76]. BGP-15 has been found to significantly improve insulin sensitivity in both insulin-resistant (ob/ob) mice and diabetic (Goto-Kakizaki [GK]) rats and insulin-resistant patients. Further, BGP-15, which does not activate PPAR, has comparable efficacy to that of rosiglitazone and metformin and produces dose-dependent insulin sensitization in diabetic GK rats and hypercholesterolemic rabbits [76]. A 4-week phase IIb clinical trial involving insulin-resistant, nondiabetic patients demonstrated that BGP-15 can significantly increase whole-body insulin sensitivity and glucose utilization. Moreover, the treatment efficacy was comparable to that associated with 12-13 weeks of rosiglitazone treatment, and BGP-15 was well tolerated with a favorable safety profile. BGP-15 was found to interact directly with heat shock factor 1 (HSF-1) and stabilize the binding of HSF-1 to its DNA response element [77]. Mechanistic studies have shown that BGP-15 functions by inhibiting c-Jun amino-terminal kinase (JNK) phosphorylation and stimulating and upregulating the HSP (HSP90 and HSP72) and NOS (constitutive NOS and neuronal NOS) systems that are decreased or deficient in people suffering from IR or DM [78–82].

The inducible HSP72 is the most abundant of all HSPs, accounting for 1-2% of cellular protein, and is rapidly induced during cellular stress. Intracellular levels of HSP72 and HSP90 have been found to correlate with whole-body glucose utilization. The induced HSPs enhance insulin sensitivity by inhibiting the kinase cascades required for activating I*κ*B kinase (IKK) and c-JNK, the inflammatory signaling protein that plays a vital role in the development of IR, thus preventing the phosphorylation of a key serine residue in insulin receptor substrate 1 (IRS-1) [83]. Subsequently, the insulin-sensitizing effects of BGP-15 are realized through multiple downstream pathways, including those that improve mitochondrial function, protect against hyperglycemia-induced mitochondrial damage, normalize the mitochondrial membrane potential, and prevent mitochondrial depletion and structural alteration [76, 84, 85]. As previous studies have indicated that mitochondrial dysfunction and metabolic overload are possibly the primary causes of IR, BGP-15, which is currently under further development, is a promising drug candidate for improving glycemic control and insulin sensitivity in people with T2DM.

### 5.4. 11*β*-HSD1 Inhibitors

11*β*-HSD1 oxidoreductase converts the inactive 11-keto forms of GCs (glucocorticoids), such as cortisone, to their active forms, such as cortisol and corticosterone, in metabolically active tissues, including the liver and adipose tissue. Elevated GC levels have been implicated in glucose intolerance, IR, dyslipidemia, and visceral obesity [86, 87]. 11*β*-HSD1 is upregulated in the adipose tissues of rodents and patients with metabolic syndrome [88, 89]. Animal studies have shown that adipose-specific 11*β*-HSD1 knockout mice are refractory to diet-induced obesity and show improved glucose tolerance and insulin sensitivity, whereas transgenic mice overexpressing 11*β*-HSD1 in fat cells have been found to develop glucose intolerance, IR, and dyslipidemia [90, 91]. As inhibition of 11*β*-HSD1 can decrease the levels of active GCs owing to its specific role in GC interconversion, 11*β*-HSD1 has been considered an attractive therapeutic target for the treatment of IR, T2DM, and metabolic syndrome [87, 92].

Several structurally diverse and selective 11*β*-HSD1 inhibitors, such as HIS-388, LG13, and PF-915275, have thus been designed [92, 93]. The antidiabetic activity of these inhibitors has been confirmed to be comparable to that of the classical insulin sensitizer pioglitazone in different insulin-resistant and/or diabetic rodent models [92, 94, 95], where the inhibitors significantly suppressed the levels of plasma and local tissue cortisol, plasma insulin, and fasting or postprandial blood glucose, and improved glucose intolerance and IR. Moreover, some 11*β*-HSD1 inhibitors have shown the same therapeutic efficacy in human clinical trials as well as in animal studies, with tolerable adverse events and no effects on basal cortisol homeostasis or normal sex hormone levels [96]. However, 11*β*-HSD1 inhibitors, which are promising insulin sensitizers, are still in the early stages of development, and no drugs of this class have entered phase III clinical trials. Additionally, as 11*β*-HSD1 is a bidirectional enzyme, which functions as both a reductase (major) and an oxidase (minor), a safer inhibitor that selectively inhibits 11*β*-HSD1 reductase activity would be preferable [86, 87].

### 5.5. AMPK Activators/Agonists

AMPK, a phylogenetically conserved serine/threonine kinase, is a master metabolic sensor of cellular energy status that regulates the cellular and whole-body energy balance. It is a heterotrimer consisting of a catalytic *α* subunit and regulatory *β* and *γ* subunits [97]. Several studies have suggested that AMPK is important in regulating insulin signaling, and dysregulation of the AMPK pathway plays critical roles in the development of IR and T2DM [98–100]. AMPK activators have been comprehensively reviewed previously [97]. AMPK activators, such as 5-aminoimidazole-4-carboxamide-1-*β*-d-ribofuranoside (AICAR), biguanides, and A-769662 (the first direct AMPK activator), have been found to reverse many of the metabolic defects associated with IR, whereas the absence or inhibition of AMPK exacerbates insulin insensitivity in target tissues [97, 101, 102]. These findings have made AMPK an attractive T2DM drug target during the past two decades. AMPK, which is activated by the phosphorylation of Thr 172 on the activation loop of the catalytic subunit by upstream kinases, stimulates glucose uptake in skeletal muscles and fatty acid oxidation in target tissues and reduces hepatic glucose production and output, partially by inhibiting the mTORC1 (mechanistic target of rapamycin complex 1)/STAT3 (signal transducer and activator of transcription 3)/Notch1 (Notch homolog 1, translocation-associated) signaling pathway [97]. The physiological functions of AMPK have been reviewed in depth previously [97, 103].

As AMPK is involved in numerous pathophysiological processes and implicated in many conditions, such as anemia, inflammation, and tumors, excessive systemic activation of AMPK may undoubtedly cause unwanted side effects, although most of its effects are beneficial. Therefore, in the absence of tissue-selective activation strategies or compounds with isoform specificity, developing a specific AMPK activator into a safer insulin sensitizer is extremely challenging [100]. Thus, despite being a seemingly promising target for drug development, no direct AMPK activators have entered clinical trials as insulin sensitizers yet. However, the effects of some multitarget drugs such as the antidiabetic effects of metformin and TZDs appear to be mediated, at least in part, by the direct or indirect activation of AMPK [104, 105]. Further investigation of AMPK regulation and its role in cellular metabolism may uncover new strategies for efficiently controlling the pharmacological modulation of AMPK using activators.

### 5.6. Biguanides (Metformin)

**Figure 3:**
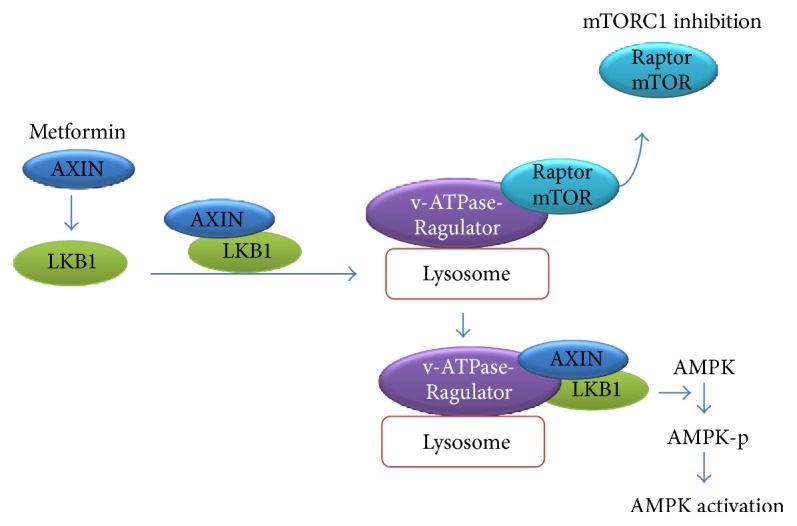
*Metformin-mediated activation of 5*′* adenosine monophosphate-activated protein kinase (AMPK) through the AXIN/LKB1-v-ATPase-Ragulator pathway*. The lysosomal v-ATPase-Ragulator complex serves as an initiating sensor for switching between AMPK and mechanistic target of rapamycin (mTOR) activation. Metformin can directly act on v-ATPase and promote the translocation of AXIN/LKB1 onto the lysosomal surface to form a complex with v-ATPase-Ragulator, thus leading to AMPK activation and the simultaneous dissociation of Raptor and mTOR, which results in the suppression of mTOR complex 1 (mTORC1) [111].

Metformin is the most representative agent of the biguanide class and the most widely prescribed antidiabetic drug to date, despite its precise mechanism of action being unknown and under active investigation. Studies indicate that metformin decreases IR and improves insulin sensitivity by facilitating the postreceptor transport of insulin and exerting positive effects on insulin receptor expression and tyrosine kinase activity, thus enhancing the insulin-mediated suppression of hepatic glucose production and insulin-stimulated glucose uptake in skeletal muscles and adipose tissues [106, 107]. At the molecular level, inhibition of complex I of the electron transport chain in mitochondria, which was once considered to be a primary target of metformin [108, 109], is believed to enhance cellular glycolysis and AMP production. However, metformin-mediated inhibition of complex I in isolated mitochondria has not been detected directly. Further, some studies, which failed to detect any change in cellular AMP levels, have nevertheless revealed that metformin can activate AMPK efficiently in primary hepatocytes and the liver of mice without affecting the energy state [110]. Moreover, Zhang et al. [111] found that metformin treatment could mimic a state of austere nutrient supply and activate AMPK and inactivate mTORC1 through the AXIN/LKB1-v-ATPase-Ragulator pathway (Figure 3), rather than as a mere consequence of disrupting metabolic processes via the inhibition of oxidative phosphorylation. The inhibition of interstitial fibrosis by metformin may also be attributable to AMPK activation and transforming growth factor *β*1 (TGF-*β*1)/Smad3 signaling suppression, which leads to the suppression of aberrant extracellular matrix (ECM) remodeling in adipose tissues and an increase in systemic insulin sensitivity [112]. As TGF-*β*1 signaling inhibits the LKB1-AMPK axis and thus facilitates the nuclear translocation of forkhead box protein O1 (FoxO1) and activation of key gluconeogenic genes, such as those of glucose-6-phosphatase (G6P) and phosphoenolpyruvate carboxykinase (PEPCK), suppression of TGF-*β*1/Smad3 signaling is linked to the suppression of hepatic gluconeogenesis [113].

Although numerous potential mechanisms have been reported for elucidating the metformin-mediated improvement in insulin sensitivity, these mechanisms uniformly indicate that AMPK activation is the central step and differ only with respect to the adaptors between metformin and AMPK activation. Metformin can thus inhibit gluconeogenesis and activate glycolysis by activating hepatic AMPK. In adipose cells, AMPK activation was shown to increase the phosphorylation of the JNK-c-Jun and mTOR-p70S6 kinase pathways and suppress the expression of phosphatase and tension homolog (PTEN), which is a negative regulator of insulin signaling [114]. Metformin also induced glucose transporter type 4 (GLUT4) translocation by increasing the phosphorylation of Cbl and expression of Cbl-associated protein (CAP) signals via AMPK activation [115]. Therefore, AMPK activation, protein kinase A (PKA) inhibition, inhibition of mitochondrial respiration, and redox stress may all play a role in the comprehensive pharmacological effect of metformin against DM. However, unless the exact binding target of metformin is identified, finding a biguanide insulin sensitizer capable of surpassing metformin in efficacy and safety will be challenging.

### 5.7. Chloroquines (CQs)

CQs, which were originally developed as antimalarial drugs, are lately being used to treat autoimmune diseases such as rheumatoid arthritis and lupus erythematosus. Interestingly, several recent studies have demonstrated their beneficial effects against both T1DM and T2DM [116]. Some reports indicate that CQ increases the binding of insulin to its receptor and potentiates insulin-mediated inhibition of hepatic gluconeogenesis [117]. Hydroxychloroquine (HCQ) has been found to significantly increase the insulin sensitivity index (ISI) and show a tendency to reduce IR (as assessed by the homeostatic model assessment for insulin resistance [HOMA-IR]) [118]. However, only a thorough understanding of the mechanisms responsible for these favorable metabolic effects, which currently remain ill-defined, may give rise to an attractive alternative therapeutic option for treating IR and DM. A clinical trial conducted on T2DM patients to investigate the metabolic effects of HCQ on blood glucose, blood pressure, and blood cholesterol was completed in 2015 and aims to contribute to the development of a new therapeutic approach to metabolic syndrome and DM (ClinicalTrials.gov identifier: NCT02026232). Similarly, a “proof-of-concept” randomized double-blinded placebo-controlled trial titled “Rediscovering Hydroxychloroquine as a Novel Insulin Sensitizer” involving insulin-resistant subjects is expected to conclude in 2017 (ClinicalTrials.gov identifier: NCT02124681). This study, which evaluates the insulin-sensitizing effects of HCQ and explores the mechanism by which HCQ affects glucose metabolism and its target organs, may determine whether HCQ can act as a potential therapeutic lead for IR disorders.

## 6. Conclusion

The critical role played by IR in the pathogenesis of T2DM and the limitations of currently available insulin sensitizers such as TZDs encourage the development of new insulin sensitizers with a higher safety profile. The recent lessons learned from developing powerful PPAR*γ* or PPAR*α*/*γ* agonists into insulin sensitizers suggest that our efforts towards finding safer insulin sensitizers may be successful only when we go beyond focusing specifically on PPAR*γ*. Moreover, several compounds with completely different modes of action have been identified in recent years. These molecules have shown promising results in early stages of development and represent diverse approaches in the search for a new generation of insulin sensitizers without TZD-like adverse effects. However, several limitations still need to be addressed, and further studies are required before these leads can be successfully developed for the clinical setting. As the identification of a potential drug target and thorough investigation of its pathophysiological functions is a long-term reiterative verification process, whether the drug targets reviewed here would lead to the development of an effective, marketable insulin sensitizer remains to be seen. Therefore, the success of any of the approaches described in this review would represent great progress, and the resulting insulin sensitizer, which would not exhibit TZD-like side effects, will undoubtedly be a valuable antidiabetic agent in the future.
